# Transformation of a post-cesarean section placental site nodule into a coexisting epithelioid trophoblastic tumor and placental site trophoblastic tumor: a case report

**DOI:** 10.1186/1746-1596-8-85

**Published:** 2013-05-20

**Authors:** Bo-Jung Chen, Chien-Jui Cheng, Wei-Yu Chen

**Affiliations:** 1 Deparment of Pathology, Taipei Medical University Hospital, Taipei, Taiwan; 2 Department of Pathology, School of Medicine, College of Medicine, Taipei Medical University, 250 Wu-Hsing St, Taipei 11031, Taiwan; 3 Department of Pathology, Wan Fang Hospital, Taipei Medical University, Taipei, Taiwan

**Keywords:** Placental site nodule, Epithelioid trophoblastic tumor, Placental site trophoblastic tumor, Intermediate trophoblast, Cesarean section

## Abstract

**Abstract**

Placental site nodules (PSNs) and epithelioid trophoblastic tumors (ETTs) respectively represent non-neoplastic and neoplastic lesions of chorionic-type intermediate trophoblasts (ITs). Many patients with a PSN have a history of a cesarean section (CS) or therapeutic abortion. Recent evidence shows that a PSN may progress to an ETT. Herein, we describe a coexisting ETT and placental site trophoblastic tumor (PSTT) intimately associated with PSNs in the post-cesarean lower uterine segment of a 41-year-old woman. The patient presented with abnormal vaginal bleeding 1 year after a cesarean delivery for her most recent pregnancy. We speculated that the neoplasms had transformed from PSNs, the formation of which was related to faulty expulsion of the placental tissue or abnormal colonization of chorionic-type ITs during the CS. Neoplastic trophoblastic cells derived from PSNs displayed differentiation plasticity toward chorionic-type ITs and implantation site ITs that were respectively constituted of an ETT and PSTT.

**Virtual slides**

The virtual slides for this article can be found here: http://www.diagnosticpathology.diagnomx.eu/vs/1597949195882123

## Background

Gestational trophoblastic neoplasms are a group of fetal trophoblastic tumors including choriocarcinomas, epithelioid trophoblastic tumors (ETTs), and placental site trophoblastic tumors (PSTTs) [1]. Recent studies demonstrated distinct trophoblastic differentiation in these tumors [1-4]. A choriocarcinoma consists of an admixture of syncytiotrophoblasts, cytotrophoblasts, and intermediate trophoblasts (ITs), whereas an ETT and PSTT respectively show differentiation of chorionic-type ITs and implantation-site ITs. Mixed gestational trophoblastic neoplasms of combinations of choriocarcinomas, ETTs, and/or PSTTs were also described [3,5-7]. The most common combination is a choriocarcinoma admixed with an ETT and/or PSTT. To our knowledge, only one case of a mixed ETT and PSTT was reported in the literature [3]. Although cellular differentiation of gestational trophoblastic neoplasms has clearly been elucidated, their pathogenesis remains poorly understood. In contrast to choriocarcinomas that commonly occur subsequent to a molar pregnancy, only a minority of cases with an ETT or PSTT had an antecedent molar pregnancy [1,4]. Little is known about the morphologic transition from a precursor lesion to a gestational trophoblastic neoplasm. Herein, we report a case of a coexisting ETT and PSTT centered in the post-cesarean lower uterine segment. Placental site nodules (PSNs) were intimately associated with the neoplasm. We speculated that the formation of these PSNs was related to a cesarean section (CS), and the PSNs subsequently progressed to the coexisting ETT and PSTT.

## Case presentation

### Clinical summary

A 41-year-old woman presented with abnormal vaginal bleeding for 2 months. She had a history of two cesarean deliveries and one spontaneous abortion. She had undergone excision of a uterine adenomyosis 3 years previous. Her latest pregnancy resulted in the delivery of a full-term baby 1 year previous. The physical examination was otherwise normal. Pelvic ultrasonography showed focal thickening of the endometrium. She received endometrial curettage with a clinical diagnosis of endometrial hyperplasia. A serum β-human chorionic gonadotropin (β-hCG) measurement was performed 1 week later because an IT tumor was suspected after pathologic examination of the endometrial curettage specimen. The β-hCG level was not elevated (1 mIU/ml; reference range: 0 ~ 5 mIU/ml). The abdominal and pelvic computed tomographic scans exhibited an enlarged uterus with an irregular contour and heterogeneous contrast enhancement without para-aortic or pelvic lymphadenopathy. No metastatic lesion was detected on a chest x-ray. She underwent a total abdominal hysterectomy. Neither postoperative chemotherapy nor radiotherapy was given. She was alive with no evidence of local recurrence or distant metastasis 30 months after the hysterectomy.

### Pathologic findings

**Figure 1:**
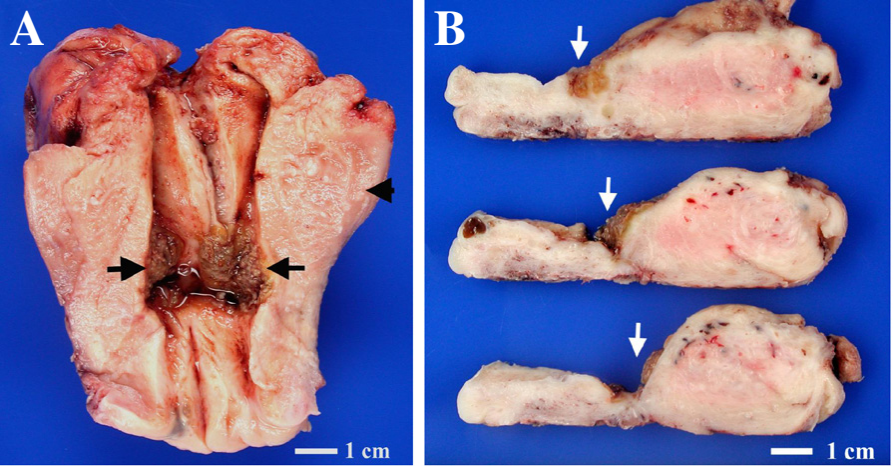
**Macroscopic features of a coexisting epithelioid trophoblastic tumor (ETT) and placental site trophoblastic tumor (PSTT) at the cesarean section site.** (**A**) An irregular, plaque-like, soft, brown tumor centered at the previous cesarean section site (arrow). Adenomyosis with thickened myometrium was noted in the myometrium (arrowhead). (**B**) Tumor invasion into the myometrium of the lower uterine segment (arrow). The uterine corpus and cervix are on the right and left, respectively.

On macroscopic examination, the uterus measured 9.0 × 8.3 × 4.2 cm and was slightly enlarged. The serosa showed focal fibrosis. The previous CS site of the lower uterine segment revealed a markedly thinned myometrium. There was an irregular, plaque-like, soft, brownish tumor centered at the CS site (Figure 1A, B). The tumor was 3.0 × 2.0 cm in dimensions and 0.9 cm in thickness. It had invaded the myometrium but was still confined to the uterus.

**Figure 2:**
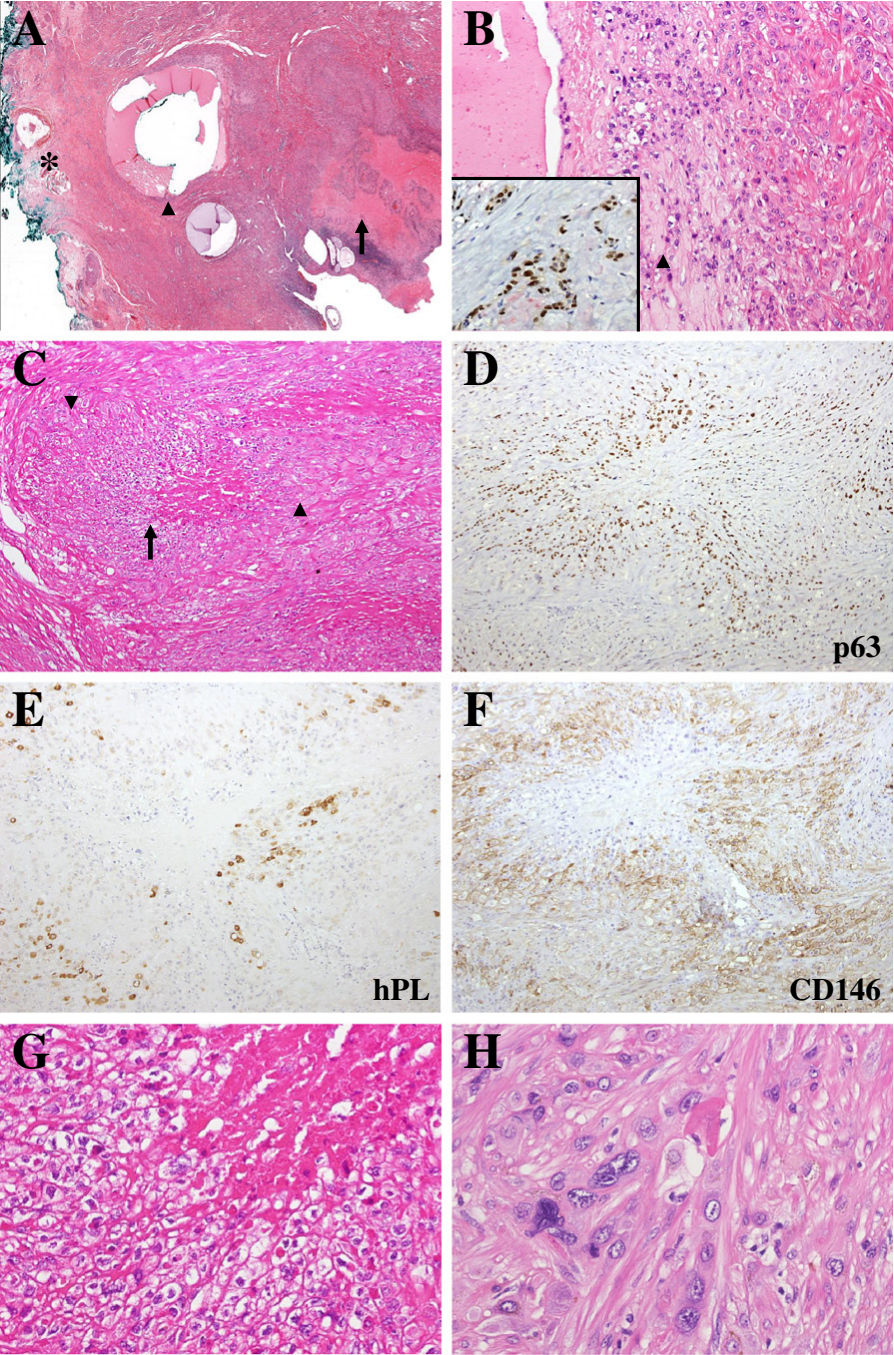
**Histologic and immunohistochemical features of a coexisting epithelioid trophoblastic tumor (ETT) and placental site trophoblastic tumor (PSTT) transformed from placental site nodules (PSNs) at the cesarean section site.** (**A**) A scanning view of the mixed ETT and PSTT shows variably sized nodules with central fibrinoid necrosis (arrow). In the deep myometrium, a suture tract, which was closely associated with the mixed ETT and PSTT, was noted (arrowhead). The tract was surrounded by PSNs. Note the suture material at the outermost surface of the lower uterine segment (asterisk). (**B**) Transformation of PSNs into the mixed ETT and PSTT. PSNs (arrowhead) located around the suture tract showed continuity with the mixed ETT and PSTT on the right. (x200) (Inset, p63 expression in PSN). (**C**) A tumor nodule composed of an ETT in the center (arrow) and a PSTT in the periphery (arrowhead) (x100). (**D**) p63 expression highlighting the ETT of the tumor nodule in Figure 2C (x100). (**E**) and (**F**) PSTT of the tumor nodule in Figure 2C demonstrated by immunoreactivity to hPL and CD146 (x100). (**G**) ETT composed of mononucleate epithelioid cells arranged in cohesive sheets. The tumor cells had distinct cell borders, clear cytoplasm, and uniform nuclei (x400). (**H**) PSTT dissecting and separating smooth muscle bundles of the myometrium (x400). (**A**, **B C**, **G**, and **H**, hematoxylin-eosin).

Histologically, the tumor consisted of variably sized nodules with central fibrinoid necrosis in the endometrium and myometrium of the lower uterine segment (Figure 2A). Suture material and suture tracts due to the previous CS were observed. The nodules commonly revealed an infiltrating border in the myometrium. The center of the nodules was composed of mononucleate epithelioid cells arranged in cohesive sheets (Figure 2C, G). The background contained hyaline extracellular matrix. The tumor cells had distinct cell borders, clear cytoplasm, and relatively uniform round nuclei with fine chromatin. Enlarged hyperchromatic nuclei were occasionally found. Mitotic figures were infrequent. Epithelioid clear cells revealed diffuse and strongly positive immunoreactivity to pan-cytokeratin (clone AE1/AE3, 1: 400; Dako, Carpentaria, CA, USA), p63 (clone 4A4, 1: 100; Santa Cruz Biotechnology, Santa Cruz, CA, USA), and HLA-G (a gift of Dr. Ie-Ming Shih) (Figure 2D). Human placental lactogen (hPL) (1: 250; Pierce Biotechnology, Rockford, IL, USA) and CD146 (1: 50; Pierce Biotechnology) were negative (Figure 2E, F). No cell immunoreactive with hCG (1: 300; Dako) was found. Ki-67 (clone MIB-1, 1:50; Dako) labeled 12% of tumor cells. The morphologic and immunohistochemical features of the epithelioid cells were characteristic of an ETT.

In the periphery of the nodules, larger pleomorphic cells, which histologically and immunohistochemically differed from tumor cells of the ETT, were seen (Figure 2C, H). They were polygonal with abundant eosinophilic cytoplasm and relatively obvious nuclear atypia. They had invaded the myometrium in cell cords, dissecting and separating the smooth muscle bundles. They were immunoreactive for CD146 and hPL but negative for p63 (Figure 2D-F). Ki-67 labeled about 10% of the tumor cells. These histologic and immunohistochemical findings were consistent with features of a PSTT. At the interface of the ETT and PSTT components, both type of tumor cells merged imperceptibly.

In addition, several microscopic hypocellular nodular lesions, closely associated with the mixed ETT and PSTT, were found in the endometrium of the lower uterine segment, the surface of the endocervix, and the fibrous scar of the CS (Figure 2B). They had circumscribed margins and were round or plaque-like in shape. They were composed of mononucleate ovoid cells arranged in single cells and small sheets in a hyaline background. These cells had uniform nuclei with fine chromatin. The immunohistochemical findings were the same to those of the ETT. Compared to the ETT, the nodular lesions were smaller in size and paucicellular. No necrosis was found. The Ki-67 proliferation index was <5%. Based on the histologic and immunohistochemical features, the smaller nodular lesions represented PSNs. In summary, the tumor represented a rare example of a coexisting ETT and PSTT that had likely transformed from PSNs. Microscopic findings of the endometrial curettage specimen were similar to those of the hysterectomy specimen, except that no PSNs were present in the former.

## Discussion

ETTs and PSTTs are rare gestational trophoblastic neoplasms, with respective differentiation toward chorionic-type ITs and implantation-site ITs [1]. The non-neoplastic counterpart of an ETT is a PSN, whereas that of a PSTT is an exaggerated placental-site reaction (EPSR). The pathogenesis of ETTs and PSTTs remains obscure. A recent study demonstrated a lack of a biologic link between an EPSR and PSTTs [8]. However, atypical PSNs with morphologic features intermediate between typical PSNs and ETTs were described [4,9]. Transformation of a PSN through an atypical PSN to a malignant ETT was also reported [10]. In a clinicopathologic study of 14 cases of ETTs, two cases were intimately associated with PSNs [3]. Those findings suggest that PSNs have the potential to develop into ETTs. The transition from a PSN to a coexisting ETT and PSTT in the current case further supports the concept. Moreover, a similar distribution of ETTs and PSNs in the uterus also implies a link between them. Approximately half of ETT cases and 40% of PSN cases are located in the lower uterine segment and/or upper endocervix [3,4,9,11,12]. A recent study showed a lack of a Y-chromosome complement in >80% of cases of ETTs [13]. Analysis of the Y allele in PSNs may be helpful to further clarify the relationship between ETTs and PSNs.

Normal placental-site tissue is expelled from the uterus within several weeks postpartum [11]. Factors contributing to the retention of ITs that lead to PSN formation remain unclear. Much evidence supports surgical interventions, including CSs and therapeutic abortions, being related to PSN formation. Many patients with PSNs have a history of a therapeutic abortion and/or CS [4,11]. Fistulous tracts of the lower uterine segment, coated by ITs resembling PSNs, were described in patients who had received a CS [14-17]. The endometrium damaged by a CS or therapeutic abortion might not undergo cyclic shedding during subsequent menstrual cycles. Perturbation of endometrial shedding may contribute to PSN formation [12]. The present case showed distribution of PSNs at the CS site, which supports the relationship between PSN formation and surgical interventions.

Interestingly, our case showed mixed features of an ETT and PSTT. The frequency of ETTs combined with other gestational trophoblastic neoplasms is not low. In fact, the histologic, ultrastructural, and immunohistochemical features of ETTs were first described in lungs of patients with a choriocarcinoma following intensive chemotherapy [18,19]. The largest clinicopathologic study of ETTs, that included 14 patients, showed three cases combined with a choriocarcinoma and/or a PSTT [3]. Only one case of a mixed ETT and PSTT without a choriocarcinoma component was found in the literature [3]. The 15-year-old patient had a history of a preceding complete mole 1 year before the diagnosis of the mixed ETT and PSTT. Abnormal vaginal bleeding was the presenting symptom. The serum β-HCG level was not available. The tumor was limited to the uterus. She underwent a hysterectomy without postoperative chemotherapy and had no evidence of tumor recurrence or metastasis 84 months later. The two cases of a mixed ETT and PSTT, including ours, had clinical presentations similar to those of a pure PSTT or ETT [3,7,20,21]. With a pure PSTT or ETT, abnormal vaginal bleeding is the most common presenting symptom, and serum β-HCG levels are elevated in approximately 80% of patients. Because only two cases of a mixed ETT and PSTT were reported, it is difficult to establish the prognostic factors of the tumor. These two cases, both of which had a tumor limited to the uterus, like most patients with a stage I pure ETT or PSTT, had a favorable prognosis after a hysterectomy. The clinical behavior of a mixed ETT and PSTT seems similar to that of a pure ETT or PSTT, but more cases are required to elucidate its prognosis. Long-term follow-up is essential for patients with a mixed ETT and PSTT, because patients with a stage I pure ETT or PSTT occasionally have local recurrence or distant metastasis during the follow-up period [3,7,20,21]. As the β-HCG level was not elevated in 20% of patients with a PSTT or ETT, glypican 3 was recently suggested to be a potential serum tumor marker of gestational trophoblastic neoplasms [22]. The role of glypican 3 as a tumor marker needs further clinical evaluation.

The hybrid features of the present tumor were consistent with the proposed model of pathogenesis of gestational trophoblastic neoplasms [2,4]. In this model, transformed trophoblastic stem cells retain differentiation plasticity. Stem cells are able to differentiate toward cytotrophoblast, syncytiotrophoblast, chorionic-type ITs and implantation-site ITs. This model also explains the sensitivity of trophoblastic neoplasms to chemotherapy. Choriocarcinomas, the most primitive trophoblastic tumors, are sensitive to chemotherapy. Conversely, ETTs and PSTTs are not responsive to chemotherapy due to their more-differentiated natures. Because of the differentiation plasticity of neoplastic trophoblasts, it is clinically critical for pathologists to thoroughly examine pathologic specimens, particularly small curettage samples, to identify components of trophoblastic neoplasms: ETTs, PSTTs, choriocarcinomas, or their combinations. Therapeutic approaches to gestational trophoblastic tumors rather depend on the differentiation of neoplastic trophoblasts. An immunohistochemical panel including cytokeratin 18, HLA-G, HSD3B1, β-HCG, β-catenin, hPL, CD146, p63, and Ki-67 can be of great help in establishing a correct diagnosis [2].

## Conclusion

This report supports PSNs possibly being precursor lesions of trophoblastic neoplasms. The present case showed that PSNs had transformed into rare gestational trophoblastic neoplasms which were composed of ETTs and PSTTs. Although PSNs are non-neoplastic lesions of chorionic-type ITs, some trophoblasts of PSNs still retain differentiation plasticity and can differentiate into other trophoblasts such as implantation-site ITs in the present tumor. The intimate association between PSNs and the CS scar indicates that surgical interventions may lead to retention of ITs in the uterus. The present case represents an extremely rare complication of a CS.

## Consent

Written informed consent was obtained from the patient for publication of this Case Report and all accompanying images. A copy of the written consent is available for review by the Editor-in-Chief of this journal.

## Abbreviations

β-hCG: β-Human chorionic gonadotropin; CS: Cesarean section; EPSR: Exaggerated placental site reaction; ETT: Epithelioid trophoblastic tumor; IT: Intermediate trophoblast; PSN: Placental-site nodule; PSTT: Placental-site trophoblastic tumor.

## Competing interests

The authors declare that they have no competing interests.

## Authors’ contribution

BJC participated in drafting the manuscript and reviewing the literature. CJC and WYC were responsible for making the pathologic diagnosis. WYC proposed the idea and revised the manuscript. All authors read and approved the final manuscript.

## References

[B1] Shih IeM Gestational trophoblastic neoplasia-pathogenesis and potential therapeutic targets Lancet Oncol 2007 8 642 650 10.1016/S1470-2045(07)70204-8 17613426

[B2] Mao TL Kurman RJ Huang CC Lin MC Shih IeM Immunohistochemistry of choriocarcinoma: an aid in differential diagnosis and in elucidating pathogenesis Am J Surg Pathol 2007 3 1726 1732 18059230 10.1097/PAS.0b013e318058a529

[B3] Shih IM Kurman RJ Epithelioid trophoblastic tumor: a neoplasm distinct from choriocarcinoma and placental site trophoblastic tumor simulating carcinoma Am J Surg Pathol 1998 22 1393 1403 10.1097/00000478-199811000-00010 9808132

[B4] Shih IM Kurman RJ The pathology of intermediate trophoblastic tumors and tumor-like lesions Int J Gynecol Pathol 2001 20 31 47 10.1097/00004347-200101000-00004 11192071

[B5] Ramondetta LM Silva EG Levenback CF Burke TW Mixed choriocarcinoma in a postmenopausal patient Int J Gynecol Cancer 2002 12 312 316 10.1046/j.1525-1438.2002.01110.x 12060455

[B6] Shen DH Khoo US Ngan HY Ng TY Chau MT Xue WC Cheung AN Coexisting epithelioid trophoblastic tumor and choriocarcinoma of the uterus following a chemoresistant hydatidiform mole Arch Pathol Lab Med 2003 127 e291 e293 12823059 10.5858/2003-127-e291-CETTAC

[B7] Baergen RN Rutgers JL Young RH Osann K Scully RE Placental site trophoblastic tumor: a study of 55 cases and review of the literature emphasizing factors of prognostic significance Gynecol Oncol 2006 100 511 520 10.1016/j.ygyno.2005.08.058 16246400

[B8] Dotto J Hui P Lack of genetic association between exaggerated placental site reaction and placental site trophoblastic tumor Int J Gynecol Pathol 2008 27 562 567 10.1097/PGP.0b013e31816d1d00 18753963

[B9] Mao TL Seidman JD Kurman RJ Shih IeM Cyclin E and p16 immunoreactivity in epithelioid trophoblastic tumor-an aid in differential diagnosis Am J Surg Pathol 2006 30 1105 1110 16931955 10.1097/01.pas.0000209854.28282.87

[B10] Tsai HW Lin CP Chou CY Li CF Chow NH Shih IM Ho CL **Placental site nodule transformed into a malignant epithelioid trophoblastic tumour with pelvic lymph node and lung metastas**is Histopathology 2008 53 601 604 18983471 10.1111/j.1365-2559.2008.03145.x

[B11] Young RH Kurman RJ Scully RE P**lacental site nodules and plaques. A clinicopathologic analysis of 20 cases** Am J Surg Pathol 1990 14 1001 1009 10.1097/00000478-199011000-00002 2240354

[B12] Huettner PC Gersell DJ Placental site nodules: a clinicopathologic study of 38 cases Int J Gynecol Pathol 1994 13 191 198 10.1097/00004347-199407000-00001 7523321

[B13] Yap KL Hafez MJ Mao TL Kurman RJ Murphy KM Shih IeM Lack of a y-chromosomal complement in the majority of gestational trophoblastic neoplasms J Oncol 2010 2010 364508 20182630 10.1155/2010/364508 PMC2825661

[B14] Ismail SM Lewis CG Shaw RW Postcaesarean section uterovesicle fistula lined by persistent intermediate trophoblast Am J Surg Pathol 1995 19 1440 1443 10.1097/00000478-199512000-00011 7503366

[B15] Fischer RJ Symptomatic cesarean scar diverticulum: a case report J Reprod Med 2006 51 742 744 17039709

[B16] O’Neill CJ Cook I McCluggage WG Postcesarean delivery uterine diffuse intermediate trophoblastic lesion resembling placental site plaque Hum Pathol 2009 40 1358 1360 10.1016/j.humpath.2009.01.023 19454357

[B17] Liang Y Zhou F Chen X Zhang X Lü B Atypical epithelioid trophoblastic lesion with cyst and fistula formation after a cesarean section: a rare form of gestational trophoblastic disease Int J Gynecol Pathol 2012 31 458 462 10.1097/PGP.0b013e31824a1dc9 22833087

[B18] Mazur MT Metastatic gestational choriocarcinoma. Unusual pathologic variant following therapy Cancer 1989 63 1370 1377 10.1002/1097-0142(19890401)63:7<1370::AID-CNCR2820630723>3.0.CO;2-G 2465817

[B19] Jones WB Romain K Erlandson RA Burt ME Lewis JL Jr Thoracotomy in the management of gestational choriocarcinoma A clinicopathologic study Cancer 1993 72 2175 2181 10.1002/1097-0142(19931001)72:7<2175::AID-CNCR2820720718>3.0.CO;2-K 8374875

[B20] Hassadia A Gillespie A Tidy J Everard RGNJ Wells M Coleman R Hancock B Placental site trophoblastic tumour: clinical features and management Gynecol Oncol 2005 99 603 607 10.1016/j.ygyno.2005.06.054 16085293

[B21] Hyman DM Bakios L Gualtiere G Carr C Grisham RN Makker V Sonoda Y Aghajanian C Jewell EL Placental site trophoblastic tumor: analysis of presentation, treatment, and outcome Gynecol Oncol 2013 129 58 62 10.1016/j.ygyno.2012.12.029 23274560

[B22] Ou-Yang RJ Hui P Yang XJ Zynger DL Expression of glypican 3 in placental site trophoblastic tumor Diagn Pathol 2010 5 64 10.1186/1746-1596-5-64 20868507 PMC2954974

